# The effects of L-carnitine on renal function and gene expression of caspase-9 and Bcl-2 in monosodium glutamate‐induced rats

**DOI:** 10.1186/s12882-021-02364-4

**Published:** 2021-05-02

**Authors:** Farhad Koohpeyma, Morvarid Siri, Shaghayegh Allahyari, Marzieh Mahmoodi, Forough Saki, Sanaz Dastghaib

**Affiliations:** 1 Shiraz Endocrinology and Metabolism Research Center, Shiraz University of Medical Sciences, 719363-5899, Shiraz, Iran; 2 Autophagy Research Center, Shiraz University of Medical Sciences, Shiraz, Iran; 3 Department of Genetic, Arsanjan Branch, Islamic Azad University, Fars, Iran; 4 Department of Clinical Nutrition, School of Nutrition and Food Sciences, Shiraz University of Medical Sciences, Shiraz, Iran

**Keywords:** L-carnitine, Kidney, Caspase-9, Bcl-2, MSG, Anti‐oxidant

## Abstract

**Background:**

Monosodium glutamate (MSG) is frequently consumed as a flavor enhancer or food additive. Possible damages induced by MSG effects on some organs have been stated in experimental animal models. The aim of the present study was to evaluate the protective effects of L-carnitine (L-ca) on the renal tissue in MSG-Induced Rats.

**Methods:**

In this regard, 60 male rats were randomly divided into six groups (n = 10/each): 1 (Control); 2 (sham); 3 (L-carnitine 200 mg/kg b.w); 4 (MSG 3 g/kg b.w); 5 (MSG + L-carnitine 100 mg/kg); and 6 (MSG + L-carnitine 200 mg/kg). After 6 months, the rats were sacrificed, the blood sample collected and the kidneys harvested for evaluation of biochemical analytes, genes expression, and histopathological changes.

**Results:**

MSG significantly increased the serum level of MDA, BUN, creatinine, uric acid and renal Caspase-9, NGAL and KIM-1 expression, but it decreased the serum activity also renal expression of SOD, catalase, GPX, and Bcl-2 expression compared to the control group. Treatment with L-ca significantly reduced the serum BUN, creatinine, uric acid and MDA level and increased catalase, GPX and SOD compared to the MSG group. However, only administration of L-ca 200 significantly decreased the caspase-9, NGAL and KIM-1; also, it increased the Bcl-2 expression in the kidney compared to the MSG group.

**Conclusions:**

Our findings indicated that L-carnitine had a major impact on the cell protection and might be an effective therapy in ameliorating the complications of the kidney induced by MSG via its antioxidant and anti-apoptotic properties.

## Background

Monosodium glutamate (MSG) as a salt form of glutamic acid [1] is used as flavoring in cooking to enhance the palatability and food selection in a meal [2]. Although MSG has proven its value as an enhancer of flavor and popular food additive, several studies have indicated possible toxic effects on the induction of metabolic changes, which can lead to major disturbances including obesity, hepatic damages, CNS (central nervous system), and reproductive disorders. [1]. Induction of oxidative stress after administration of MSG is one of these disturbances [3–5]. In this view, increased oxidative stress has been found to enhance hypercholesterolemia, hypertension, diabetes mellitus, and chronic renal failure [6, 7]. Also, it has been shown that renal damage is related to MSG induced oxidative stress as the main cause of kidney injury [8]. In the cells, the imbalance between production/elimination of free radicals (oxygen radicals and other reactive oxygen species (ROS)) leads to oxidative stress which causes detrimental damages to the macromolecules and vital structures in biological systems [9, 10]. Several intracellular and extracellular factors such as diet, environmental factors, hormones, cytokines, and detoxification processes contribute to the oxidative stress [11].

Additionally, the decreased antioxidant defense system is characterized by reduced levels of anti-oxidant enzymes; also, enhanced lipid peroxidation has been reported in MSG-exposed rats [12–14]. Moreover, oxidative stress makes the kidney tissue susceptible to damage by various alterations including the promotion of lipid peroxidation, protein modification, and DNA damage, leading to cell death [14]. It is now widely accepted that oxidative stress can lead to changes in the expression of specific genes including renal injury-related genes, like kidney injury molecule 1 (KIM-1), tumor necrosis factor alpha (TNF-α), and Neutrophil gelatinase-associated lipocalin protein (NGAL) [15]. Under kidney tubular dysfunction for any reasons like oxidative stress and inflammation, NGAL, as a member of lipocalin superfamily, is overexpressed and secreted in the urine [16]. In normal conditions, NGAL is expressed and secreted by the kidneys, stomach, lungs, and colon at a very low level, and completely reabsorbed after glomerular filtration [17]. KIM-1, as a specific and sensitive biomarker in the diagnosis of kidney injury, plays an important role in removing the cellular debris from the tubular lumen in renal injury. KIM-1 is expressed at low levels in normal kidney tissues [18].

Antioxidants act as free radical scavengers that help to overcome deteriorating effects of ROS. In this perspective, it seems that the use of potential antioxidants has been effective to reverse these changes induced by MSG. L-carnitine is an amino acid; approximately, 25 % is synthesized from lysine and methionine and naturally produced in the body; it is a water-soluble antioxidant mostly derived from the human diet [19]. It has been shown that carnitine and its acyl derivatives had potential antioxidant effects [20]. Also, carnitine had beneficial effects on coronary heart disease, and heart and renal failure characterized by enhanced oxidative stress [20, 21]. Moreover, carnitine ameliorates the oxidative tissue damage due to its free radical scavenging and antioxidant effects [20, 21]. On the other hand, oxidative stress leads to an increase in the release of cytochrome C from the mitochondria which activates the apoptotic mechanism in the cells by activating caspases (caspase-3/7, 8 and 9) and pushing the cells to the programmed cell death. Expression of the Bcl-2 protein family prevents the induction of apoptosis by a variety of oxidative stresses, including ionizing radiation, heat shock, or inhibition of GSH synthesis. It has been suggested that the Bcl-2 gene product inhibits apoptosis by interacting with mitochondrial Super oxide dismutase (SOD) [22]. Based on previous investigations, L-carnitine can decrease the cells apoptosis through inhibiting the activity of caspases, ameliorating oxidative stress, and enhancing the antioxidant defense systems in various tissues [23, 24].

Therefore, the aim of the present study was to evaluate the effects of L-carnitine on histopathological changes of the renal tissue, anti-oxidant activity, and gene expression of Caspase-9 and Bcl-2 in Monosodium Glutamate-Induced Rats.

## Materials and methods

### Experimental animals

In the current study, 60 adult male spargue-dawley rats (10 weeks old) weighting 250 ± 20 g were purchased from the animal laboratory center of Shiraz University of Medical Sciences to evaluate the effect of MSG and L-Ca. One week prior to the study, they were acclimatized to the thermal conditions and the animal laboratory facilities. They were housed in standard cages, five per cage, with 12/12 hours’ light-dark cycles at temperature of 23 ± 2 °C.

### Ethics committee approval

This study protocol was approved by the Ethics Committee of Shiraz University of Medical Sciences and performed in accordance with the Ethical Standards laid down in the 1964 Declaration of Helsinki and its later amendments. It is in line with the ARRIVE (Animal Research: Reporting of in Vivo Experiments) guidelines about using and coring of research animals.

### Experimental design

The rats were divided simple randomly into six groups of 10 rats per group (according to Hassan et al., 2014) [25]. They were Group 1: the control group (without treatment), group 2: the sham group, normal saline (solvent of the MSG and L-carnitine), group 3: the healthy group (L-Ca 200) (received the L-carnitine 200 mg/kg b.w), group 4: MSG 3 g/kg b. w. day, group 5: MSG + L-carnitine 100 mg/kg b.w.day, and group 6: MSG + L-carnitine 200 mg/kg b.w.day. The rats were given MSG and L-carnitine by oral gavage once a day at 8:00 am for 6 months.

Several studies have shown that ingestion of MSG at a dose of 3 g/kg b.w. induces oxidative damage in the rats [25]. Therefore, we applied the identical dose.

At the end of the study, the rats were under anesthesia with ketamine (10 %)/ xylazine (2 %) mixture (80/5 mg/kg) (Alfasan, Netherland), and 5 ml blood was collected by cardiac puncture; the samples were centrifuged at 3500 rpm for 12 min to separate the serum and stored at -70ºc for further biochemical parameters analysis. Eventually, the animals were sacrificed by sodium thiopental intraperitoneally (100 mg/kg) [26] and then the kidney tissues were removed.

### Determination of serum biochemical parameters

Serum BUN (mg/dl), creatinine(mg/dl), uric acid(mg/dl), Ca (mg/dl), P(mg/dl) and total protein (g/dl) were measured at the end of the study by an enzymatic colorimetric assays with a DIRUI (CS-T240, China) auto-clinical chemistry-analyzer and commercial diagnostic kit Biosystem company, Spain. Serum MDA (nmol/ml), GPX (IU/ml), catalase (IU/ml), and SOD (IU/ml) were measured with ELISA method produced by ZelBio GmbH Germany following the manufacturer^’^s recommendations.

### RNA isolation and quantitative RT-PCR gene expression levels

The total RNA from the kidney tissue was isolated using the TRIzol reagent (Invitrogen) [27], and the cDNA was synthesized following the manufacturer’s protocol, using 1 µg RNA (Prime ScriptTM RT reagent Kit, Takara). RT-PCR was done using a standard SYBR-green PCR kit (SYBR Premix EX TaqTM II, Takara), and the gene-specific PCR amplification was conducted using the Applied Biosystems StepOnePlus™ Real-Time PCR System (Applied Biosystems, USA). Primer sequences are demonstrated in Table 1. All experiments were performed in quadruplicate. Relative expression was determined by the 2^−ΔΔCt^ method using the housekeeping gene, GAPDH, as an internal control, and the fold change was calculated by its comparison with the corresponding control group [28].

**Table 1:** Gene specific-forward and reverse primer sequences

Primer	Sequences (5’->3’)	PCR Product length
*Cas9: F*	ACATCTTCAATGGGACCGGC	85 bp
*Cas9: R*	TCTTTCTGCTCACCACCACAG	
*GAPDH: F*	AAAGAGATGCTGAACGGGCA	100 bp
*GAPDH: R*	ACAAGGGAAACTTGTCCACGA	
*Bcl-2: F*	GGAGGATTGTGGCCTTCTTT	100 bp
*Bcl-2: R*	GTCATCCACAGAGCGATGTT	
*Ngal: F*	TGAACTGAAGGAGCGATTCG	86 bp
*Ngal: R*	ATTGGTCGGTGGGAACAGA	
*Kim-1: F*	ACTCCTGCAGACTGGAATGG	214 bp
*Kim-1: R*	CAAAGCTCAGAGAGCCCATC	
*CAT: F* *CAT: R*	GCGAATGGAGAGGCAGTGTA GAGTGACGTTGTCTTCATTAGCACTG	625 bp
*GPx: F* *GPx: R*	CTCTCCGCGGTGGCACAGT CCACCACCGGGTCGGACATAC	290 bp
*Cu-Zn SOD: F*	GCAGAAGGCAAGCGGTGAAC	447 bp
*Cu-Zn SOD: R*	TAGCAGGACAGCAGATGAGT	

### Renal histopathology

The collected tissues were processed and sectioned at a thickness of 5 μm and stained with trichrome masson [29]. The sections were then dehydrated, cleared, and eventually mounted in entellane (Merck Co., Germany); they were then cover-slipped. The prepared slides were examined under light microscopy (Olympus, Japan) at 4- 40X [30].

### Statistical analysis

The data were represented as Mean ± SEM. Statistical analysis was performed using IBM SPSS for Windows (version 22, SPSS Inc., Chicago, IL, USA). The values were compared using one-way analysis of variance (ANOVA) or Tukey test. The significance level was considered as *P* < 0.05.

## Results

### Evaluation of the effects of L-carnitine on renal function parameters

**Fig. 1:**
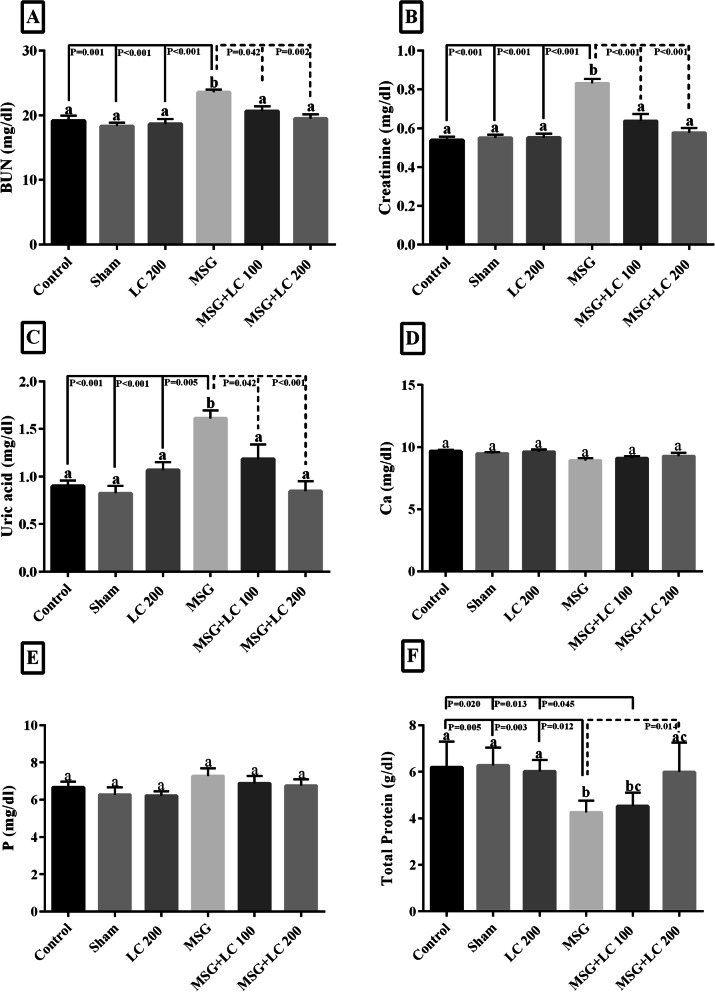
Serum biochemical parameters measured after 6 months in the experimental groups. **a** BUN, **b** creatinine, **c** uric acid, **d** Ca, **e** P, **f** Total protein was measured using an enzymatic colorimetric method. Control without any treatment, sham treated with normal saline (solvent of the Monosodium Glutamate and L-carnitine), L-ca 200 received the L-carnitine 200 mg/kg b.w, MSG treated with 3 g/kg b. w. day, MSG-LC100 received 3 g/kg MSG and 100 mg/kg L-carnitine b.w.day, MSG-LC200 received 3 g/kg MSG and 200 mg/kg L-carnitine b.w.day for 6 months. Data are expressed as Mean ± SEM, in each graph. There were no significant differences between the columns containing at least one similar letter. However, different letters reveal a significant difference (*p* < 0.05)

As displayed in Fig. 1a-c, MSG significantly increased the BUN, creatinine and uric acid compared to the control group. Treatment with L-ca 100 and L-ca 200 significantly decreased the BUN, creatinine and uric acid in comparison to the MSG group. On the other hand, the serum total protein significantly decreased in the MSG-treated group and L-ca 200 could increase it without any change in the control group. As shown in Fig. 1f the amount of serum total protein significantly decreased in MSG rats while L-ca 200 increase it as well as control group.

### Evaluation of the effects of L-carnitine on serum calcium, phosphorous concentrations

No significant changes were seen in the calcium and phosphorous concentration in all treated groups compared to both control and MSG groups (Fig. 1d and e).

### Evaluation of the antioxidant properties of L-carnitine

MDA, as an indicator of lipid peroxidation and oxidative stress, was significantly increased in the MSG group (*p* < 0.05) compared to the control group. After treatment, MDA was significantly decreased in the L-ca 100 (*p* < 0.05) and L-ca 200 (*p* < 0.05) groups. On the other hand, MSG significantly declined in the GPX, catalase and SOD activity in comparison with the controls, while L-carnitine significantly dealt with this effect and improved the anti-oxidant enzyme activity (Table 2).

**Table 2:** Evaluation of serum malondialdehyde (MDA), catalase, glutathione peroxidase (GPX), super oxide dismutase (SOD) in different experimental groups

Parameters Groups	MDA (nmol/ml)	Catalase (IU/ml)	GPX (IU/ml)	SOD (IU/ml)
**Control**	2.55 ± 1.30^a^	38.12 ± 2.44^a^	432.6 ± 33.9^a^	2.07 ± 0.9^a^
**Sham**	2.01 ± 0.74^a^	39.90 ± 1.78^a^	433.59 ± 19.9^a^	1.87 ± 0.7^a^
**L-ca 200**	2.16 ± 0.89^a^	40.30 ± 2.51^a^	418.30 ± 37.7^a^	2.02 ± 0.2^a^
**SMG**	5.54 ± 0.83^b^	20.75 ± 2.33^b^	297.22 ± 31.4^b^	0.51 ± 0.03^b^
**SMG + L-ca100**	2.90 ± 1.08^a^	38.36 ± 6.54^a^	409.0 ± 38.2^a^	1.55 ± 0.5^ab^
**SMG + L-ca200**	2.26 ± 1.01^a^	40.20 ± 6.23^a^	428.6 ± 60.6^a^	1.77 ± 0.7^a^

^a,b,c^There was no significant difference between columns, which have at least one similar letter. However, dissimilar letters indicate a significant difference (*p* < 0.05)

### Evaluation of L-carnitine effects on the expression levels of renal antioxidant enzymes catalase, GPx, SOD

**Fig. 2:**
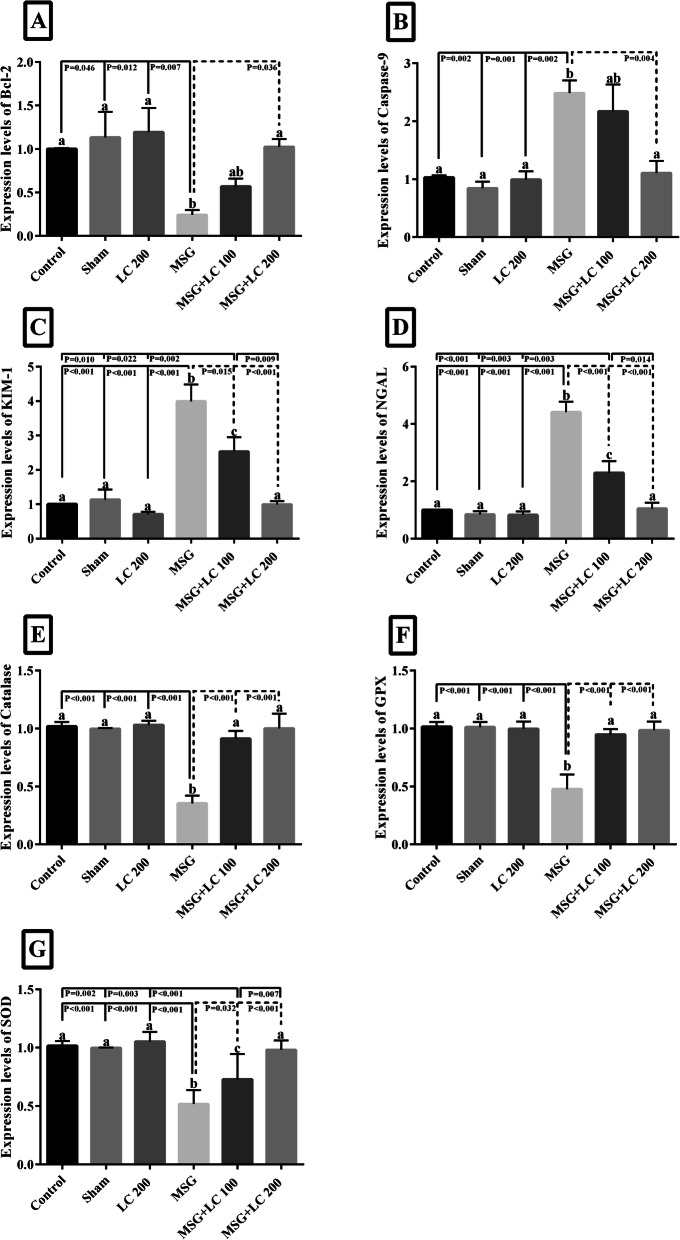
mRNA expression status in different experimental groups. **a** Bcl-2, **b** Caspase-9, **c** KIM-1, **d** NGAL (**e**) catalase, **f** GPx, and **g** Cu-Zn SOD mRNA expressions were measured by real-time PCR in the Control without any treatment, sham treated with normal saline (solvent of the Monosodium Glutamate and L-carnitine), L-ca 200 received the L-carnitine 200 mg/kg b.w, MSG treated with 3 g/kg b. w. day, MSG-LC100 received 3 g/kg MSG and 100 mg/kg L-carnitine b.w.day, MSG-LC200 received 3 g/kg MSG and 200 mg/kg L-carnitine b.w. day for 6 months. Data are presented as Mean ± SEM. There were no significant differences between the columns containing at least one similar letter. However, different letters reveal a significant difference (*p* < 0.05)

Expression levels of catalase, GPx, and SOD significantly decreased in the MSG group compared to the control group (Fig. 2e-g). However, L-carnitine significantly enhanced the expression levels of catalase, GPx, SOD compared to the MSG group. On the other hand, treatment with L-ca 200 mg/kg increased the expression levels of catalase, GPx, and SOD compared to the MSG group and reversed its adverse effects to induce renal oxidative stress better than 100 mg/kg. (Fig. 2e-g).

### Evaluation of the effects of L-carnitine on kidney expression levels of,Bcl-2, Caspase-9, NGAL, KIM-1

Expression levels of Bcl-2 significantly decreased in the MSG group compared to the control group (Fig. 2a). However, L-ca 200 significantly enhanced the expression levels of Bcl-2 compared to the MSG group. The renal expression levels of Caspase-9, NGAL, and KIM-1 significantly increased in the MSG group compared to the control group. On the other hand, treatment with L-ca in a dose- dependent manner reduced the expression levels of caspase-9, NGAL, and KIM-1 compared to the MSG group and reversed its adverse effects. (Fig. 2b-d).

### Evaluation of the effects of L-carnitine on renal histopathological changes

**Fig. 3:**
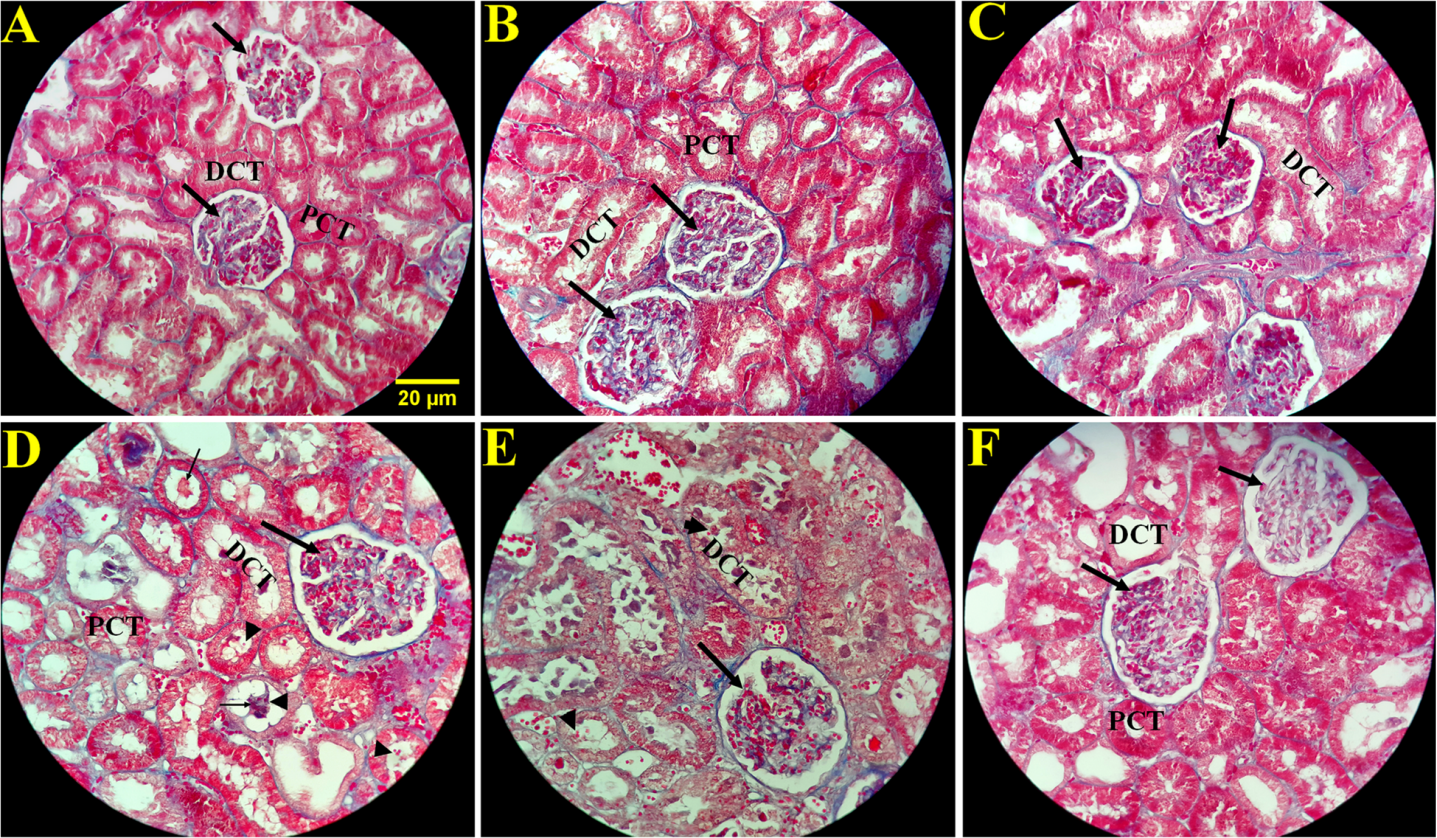
Photomicrograph of the renal histology in different groups (trichrome masson stain×400). No histopathological changes were shown in the control (**a**), sham (**b**), and L-Ca 200 (**c**) groups. In the MSG group (**d**), an increment in local inflammation, edema, interstitial hemorrhage, and Hyaline casts (thin arrow) was observed multifocally from the cortex to the inner medulla along nephrons. Some of these hyaline casts contained red blood cells, and red blood cell casts were also observed (pick arrow). Small, focal hemorrhage was scattered in the interstitium in the cortex. On the other hand, the destruction and deformation of the proximal and distal convoluted tubules were observed in some places in the MSG group. The MSG + L-ca 100 group (**e**) showed no significant changes compared to the MSG group, while the MSG + L-ca 200 group (**f**) showed significant amelioration in the MSG-induced structure damages such as inflammation, edema, interstitial hemorrhage, and deformation of proximal and distal convoluted tubules in renal tissue. PCT: proximal convoluted tubule; DCT: Distal convoluted tubule. The thick arrow indicates the glomeruli of the kidney tissue

The kidneys of the control, sham and L-ca 200 groups were completely normal and healthy in the terms of histo-architecture (Fig. 3). In the MSG group, an increment in local inflammation, edema, interstitial hemorrhage, and Hyaline casts were observed multi-focally from the cortex to inner medulla along nephrons. Some of these hyaline casts contained red blood cells; also, the red blood cell casts were observed. Small, focal hemorrhage was scattered in the interstitium in the cortex. On the other hand, the destruction and deformation of the proximal and distal convoluted tubules were observed in some places; the MSG + L-ca 100 group showed no significant histological changes compared to the MSG group (except the decrease in the inflammatory volume), but the MSG + L-ca 200 group showed ameliorated damages in the structure renal tissue.

## Discussion

To the best of our knowledge, this study was the first attempt to investigate the significant protective effect of L-carnitine consumption through anti-oxidant and anti-apoptotic properties on the renal tissue to improve damages induced by Monosodium Glutamate. Oxidative stress induces tissue damage through free radical generation and profound lipid peroxidation, as demonstrated by increasing MDA [31]. Several studies have demonstrated that MSG induces oxidative stress by the production of free radicals which could explain the pathophysiology of many disorders and dysfunctions of the tissues [32]. Different mechanisms including DNA damage, promotion of lipid peroxidation, and protein modification are caused by oxidative stress lead to tissue damages which have been implicated in the pathogenesis of various systemic diseases. One of the organs that is highly vulnerable to damage caused by free radicals is the kidney, probably due to the abundance of long-chain polyunsaturated fatty acids in the renal lipids; also, it is accepted that accumulation of ROS leads to acute and chronic renal failure [33, 34]. It has been reported that monosodium glutamate influences the tissue-specific oxidative stress in different organs, especially the liver and kidney [35]. These effects on the liver and kidney probably because these organs are mainly responsible for detoxification of input compounds in the body. Based on our biochemical tests the amount of serum total protein in MSG treated rats significantly decreased, nonetheless BUN, uric acid, and creatinine increased being in line with several studies revealed MSG induced-liver/kidney injuries [36–38].

The reduction of serum total protein in MSG treated group may be due to both liver damages and kidney dysfunction. On the other hand, renal pathological analysis showed the destructive and deformities impact of MSG on proximal and distal convoluted tubules. However, future study should determine liver function test and urinary albumin excretion to confirm the reason of reduced serum total protein in MSG treated group.

One of the vital cofactors required for transport of long-chain fatty acids into the mitochondria for production of cellular energy is carnitine (β-hydroxy-γ-N-trimethylaminobutyric acid). Beyond its carrier effects, L-carnitine also helps to eliminate the products of fatty acid metabolism and other toxic compounds from the cells [39]. The data have shown that L-carnitine supplementation significantly scavenge free radicals and protect the cells from oxidative stress [31] by decreasing the serum MDA level and increased activity of catalase, GPX, and SOD enzymes. On the other hand, Siktar et al. [40] and Canbolat et al. [41] suggested that L-carnitine intravenous infusion for 2 months could be effective in attenuating the oxidative stress responses. They reported that L-Ca enhanced the antioxidant status (increase in the serum GPX activity, total anti-oxidant capacity, and GSH/GSSG ratio) and improved the performance of the patients undergoing chronic hemodialysis therapy [39]. Several findings demonstrate that L-carnitine treatment inhibits the MDA production and reverses the depletion of GSH as well as erythrocyte antioxidant enzyme activities in CRF animals [42]. These finding are exactly in line with our results.

Additionally, accumulation of free toxic compounds such as long chain fatty acid surrounding the mitochondria induces mitochondrial membrane permeability because of mitochondrial membrane depolarization and membrane canals formation. These changes increase the release of cytochrome C from the mitochondria which activates the apoptotic mechanism in the cells by activating the caspase cascades [43]. The release of cytochrome C from the mitochondria leads to production of a caspase-activating complex of cytochrome c, APAF-1, and procaspase-9, triggers the up-regulation of caspase-9, and subsequently increases the expression of caspase-3, followed by the induction of apoptotic events in the cell [44]. It is demonstrated that overexpression of anti-apoptotic Bcl-2 inhibits the cell death by suppressing cytochrome c, which is released in response to pro-apoptotic stimuli. The level of apoptotic or anti-apoptotic Bcl-2 is a factor that plays a crucial role in determining whether or not the cells will undergo apoptosis [45]. It has been indicated that administration of MSG can induce activation of glutamate receptors which triggers the intracellular Ca^2+^ signaling pathway. When there is an excess of calcium in the organelles including the endoplasmic reticulum, nucleus, and mitochondria, calcium-dependent enzymes, including proteases and endonucleases, like caspases are activated and provide preliminaries for apoptosis [32]. It has been indicated that prolonged administration of MSG (4 mg/g) significantly induces apoptosis in a time- dependent manner in the rat’s thymocyte cells via disturbing the fine balances between prooxidant-antioxidant system, with excessive production of ROS and resulting oxidative stress status [46]. Another study obtained similar data; the researchers revealed that intraperitonially administered MSG (4 mg/g of body weight) induced oxidative stress mediated apoptosis in the thymus cells of rats [47]. However, L-carnitine supplementation ameliorates the accumulation of long chain fatty acid around the mitochondria which could inhibit the mitochondrial membrane depolarization and permeability and ultimately suppresses the cells apoptosis [48, 49]. Also, L-carnitine can decrease the cell apoptosis through inhibiting the activity of caspases, ameliorating oxidative stress, and enhancing antioxidant defense systems [49, 50]. Mutomba et al. showed that L-carnitine could inhibit the activity of an initiator caspase, caspase 8, as well as the processing of caspase 9, thereby effectively inhibiting cleavage and activation of downstream caspases. The use of a simple non-toxic metabolite like carnitine, as an anti-apoptotic agent, is very attractive compared to synthetic inhibitors with inevitable side effects [51]. Previously, Modanloo et al. reported that L-carnitine could protect the human proximal tubule epithelial HK-2 cells from H2O2-induced cell death. The results of this paper showed that mitochondrial dysfunction associated with cell apoptosis including membrane potential loss, down-regulation of Bcl-2 and up-regulation of Bax, activation of caspase-3, and the release of cytochrome c were abrogated in the presence of L-carnitine [52]. Xie et al. also showed that L-Ca decreased cytochrome c release and caspase-3 and caspase-9 activation in the serum-deprived MC3T3-E1 cells [53]. Moreover, the present study demonstrated that treatment with L-carnitine significantly reduced the gene expression of Caspase-9 and significantly increased the gene expression of Bcl-2. We evaluated the renal cell death by focusing on the RNA expression of apoptosis indicators Bcl-2, Caspase-9, and biomarkers of nephropathy (NGAL, KIM-1). It is recommended that special observations tests, covering deoxynucleotidyl transferase dUTP Nick-End-Labeling ( TUNEL ),and BAX/Bcl-2 immunoblotting required to confirm definitely the occurrence of apoptosis in renal tissue. That is could be a related limitation which is can be considered in future studies.

In the present study MSG caused a significant increase in renal expression of KIM-1 and NGAL as well as increased serum concentrations of BUN, uric acid, and creatinine. However, treatment with L-Ca in a dose dependent manner significantly reduced KIM-1 and NGAL gene expression. It has been widely accepted that early detection of renal damages is vital to prevent irreparable injuries. Novel biomarkers such as, kidney injury molecule-1 (KIM-1) and neutrophil gelatinase-associated lipocalin (NGAL) can be good indicators in the assessment of renal damages [54]. The increased expression of KIM-1 as an extracellular protein was found at very high levels on the apical membrane of proximal tubule cells after ischemic and nephrotoxic injury [55]. NGAL may eventually have prognostic value in predicting not only acute, but also chronic, worsening in renal function in patients already affected by chronic nephropathies [56]. Previously it has been reported that curcumin as an anti-oxidant reduce gene and protein expression of KIM-1 and NGAL and alleviate oxidative toxic stress in the kidney tissue of type 1 diabetes rats [17]. Another study revealed that gentamycin induced nephrotoxicity by over-producing of reactive oxygen species and free radicals and significantly increased serum NGAL, KIM-1 and cystatin-c whereas Irbesartan and other angiotensin II blockers reverse this effect and improve renal function through Modulation of oxidative stress and endogenous antioxidant Capacity [57].

The renal cyto-architecture undergoes changes with MSG through increased glomerular hyper-cellularity, inflammatory cells infiltration in the renal cortex, tubular cells edema, and eventually renal tubules degeneration. A proposed molecular mechanism of MSG-induced ROS production is that chronic MSG exposure increases the production of Glutamate which may raise the activity of one of the potential ROS generators like α-ketoglutarate dehydrogenase in the rat kidney. On the other hand, increased intracellular calcium level via glutamate receptors can lead to generation of more free radicals and subsequently a rise in lipid peroxidation. Additionally, cystine uptake inhibition leads to decreased GSH levels that may further promote ROS-mediated renal cell damage [58, 59]. On the other hand, our results indicated that administration of L-carnitine significantly ameliorated the kidney tissue damage and renal function markers such as creatinine, BUN and uric acid. It has been shown that the administration of MSG leads to kidney dysfunction, which is in the same line with our results [60]. Moreover, Vercoutere et al. [61] demonstrated that MSG resulted in several alterations in the cell lines of the kidney convoluted tubules and Bowman’s corpuscles which is associated with variations in the tubular reabsorption threshold, renal blood flow, and glomerular filtration rate. It has been shown that these changes can be attributed to the nephrotoxic effect of MSG which causes functional and cellular damage [60]. MSG increased the oxidative stress that leads to kidney tissue and function damage via several mechanisms including: (1) reduced activities of some antioxidant enzymes as catalase (CAT), superoxide dismutase (SOD), glutathione-S-transferase (GST) and glutathione (GSH) and increased MDA level in the kidney, (2) increased activity of α-ketoglutarate dehydrogenase, a potential ROS generator, (3) enhanced free radical generation through increased intracellular calcium level, and (4) decreased GSH levels that may increase ROS-induced renal cell damage [12, 14, 60]. Hence, L-carnitine prevents the MSG-induced renal damage through its potential antioxidant effects. It has also been shown that the antioxidant effects of L-carnitine are associated with scavenging free radicals, preventing reactive oxygen species (ROS) formation through maintaining mitochondria integrity in stress condition along with inhibiting ROS-generating enzymes, such as NAPDH oxidases and improving the synthesis of antioxidant enzymes including SOD, GSH, GST, and CAT [62].

## Conclusions

The present study demonstrated that L-carnitine improved the oxidative stress, renal histopathological changes and the incidence of the apoptosis induced by MSG. Above all, our current investigation highlights the potential role for the use of L-carnitine to inhibit destructive effects of MSG on the kidney tissue. Nonetheless, its relevance to human biology needs to be addressed by more investigations.

## Data Availability

The datasets used and analyzed during the current study are available from the corresponding author on reasonable request.

## Abbreviations

L-ca: L-carnitine
MSG: Monosodium Glutamate
MDA: Malondialdehyde
Ca: Calcium
P: Phosphorous
PCT: Proximal convoluted tubule
DCT: Distal convoluted tubule
ROS: Reactive oxygen species
SOD: Superoxide dismutase
BUN: Blood urea nitrogen
GPX: Glutathione peroxidase
ELISA: Enzyme linked immunosorbent assay
APAF1: Apoptotic protease activating factor 1
CAT: Catalase
